# Time-Efficient High-Rate Data Flooding in One-Dimensional Acoustic Underwater Sensor Networks

**DOI:** 10.3390/s151127671

**Published:** 2015-10-30

**Authors:** Jae Kyun Kwon, Bo-Min Seo, Kyungsu Yun, Ho-Shin Cho

**Affiliations:** 1 Department of Electronic Engineering, Yeungnam University, Gyeongsan 712-749, Korea; E-Mail: jack@yumail.ac.kr; 2 School of Electronics Engineering, Kyungpook National University, Daegu 702-701, Korea; E-Mail: bmseo@ee.knu.ac.kr; 3 Institute of Industrial Technology, Yeungnam University, Gyeongsan 712-749, Korea; E-Mail: kadbonow@ynu.ac.kr; 4 College of IT Engineering, Kyungpook National University, Daegu 702-701, Korea

**Keywords:** underwater sensor network, time efficiency, one-dimensional deployment, power consumption

## Abstract

Because underwater communication environments have poor characteristics, such as severe attenuation, large propagation delays and narrow bandwidths, data is normally transmitted at low rates through acoustic waves. On the other hand, as high traffic has recently been required in diverse areas, high rate transmission has become necessary. In this paper, transmission/reception timing schemes that maximize the time axis use efficiency to improve the resource efficiency for high rate transmission are proposed. The excellence of the proposed scheme is identified by examining the power distributions by node, rate bounds, power levels depending on the rates and number of nodes, and network split gains through mathematical analysis and numerical results. In addition, the simulation results show that the proposed scheme outperforms the existing packet train method.

## 1. Introduction

Underwater sensor networks (UWSNs) have potential applications such as oceanic environmental monitoring, tactical surveillance, and communication among autonomous unmanned vehicles (AUVs), unmanned underwater vehicles (UUVs), divers, and sensors [1,2,3]. The underwater channel characteristics are significantly different from terrestrial ones; the communication bandwidth is narrow and the propagation delay of acoustic waves is large [1,3,4]. Therefore, new protocols for medium access control (MAC) in UWSNs have been developed despite there being sufficient terrestrial MAC protocols.

The current research efforts on underwater MAC protocols have focused on increasing the channel utilization, avoiding hidden node problems, reducing delay, and enhancing fairness. First, to improve channel utilization, several underwater MAC protocols such as MACA-MN [5], MACA-UPT [6] and BiC-MAC [7], employ the packet train approach, where multiple consecutive data packets are transmitted in each round of a handshake. To address the hidden node problem, conventional underwater MAC protocols, such as Slotted-FAMA [8] and DACAP [9] have avoided the hidden node collisions by inserting a certain amount of waiting time, which results in a longer handshake exchange time and a longer delay. Recently, a way of reserving the channel through multi-hops at once is proposed, which is called cascading multi-hop reservation and transmission (CMRT) [10]. CMRT reduces the time taken for control packet exchange, and accordingly reduces the delay and increases the throughput. To enhance fairness, SF-MAC [11] has been proposed.

Although power saving and reliable transmission have been of interest because of the poor channel characteristics, the demands for enhancing the data rates are also increasing because the currently supported rates are extremely low, and higher rates will enable new applications, such as video or security data transmission [2,12,13]. To enhance the data rate under limited channel capacity, the above-mentioned packet train scheme tries to fill the time-axis by transmitting multiple packets at once without individual handshaking. Such a high usage of time is a reasonable and straightforward way to increase the data rate. In this paper, the approach of packing time-axis is adopted to enlarge the channel utilization in one-dimensionally linearly configured multi-hop sensor networks, In particular, an internode transmission/reception timing schedule is designed to minimize the idle sections on the time axis. Thereafter, the scheme is analyzed mathematically to observe the power consumption at nodes through numerical results. Because data is gathered at the sink, the transmission/reception data cumulatively increases as they become closer to the sink along with rapid increases in power consumption. This will be analyzed quantitatively. That is, this paper first optimizes the data transmission rates through the proposed internode transmission/reception timing scheme and enables an estimation of the batteries necessary according to the node by observing the minimum power levels according to the node and the minimum overall necessary power. After that, the proposed scheme is compared with the existing packet train scheme by a computer simulation. The simulation environments reflect the realistic underwater channels in terms of the distances and path loss models.

The paper is organized as follows: in Section 2, node deployment and frame timing are determined and the appropriate transmission/reception timing schemes are proposed. In Section 3, signal-to-interference-plus-noise ratio (SINR) and power consumption are derived, and the numerical results and discussion of the results are provided. The simulation results are given in Section 4, and Section 5 reports the conclusions.

## 2. Deployment and Proposed Transmission/Reception Timing Schemes

### 2.1. Deployment and Environments

**Figure 1:**
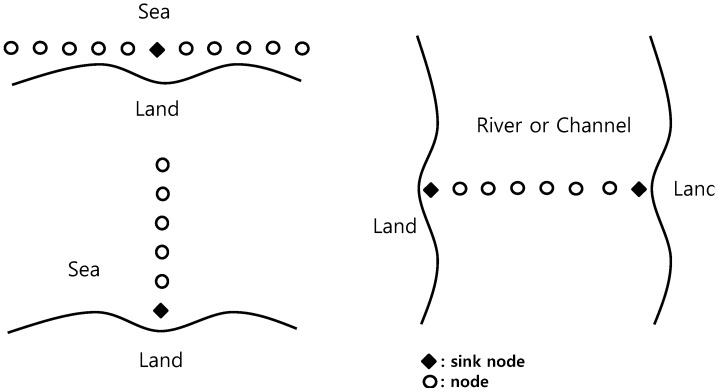
Place for the application of a one-dimensional linear sensor node deployment.

Regarding sensor node deployment, although two-dimensional or three-dimensional deployment can be generally considered, in this paper, one-dimensional linear equidistant deployment is assumed to be the easiest form and the deployment may be expanded to other complicated forms in later studies. The places for the application of this deployment may include cases where the nodes are deployed along the seaside, perpendicularly to the seaside, or across the width of a river, as shown in Figure 1. The locations of the sink nodes that gather data can be the center or both ends, but both ends become one end if the nodes are divided into two groups.

**Figure 2:**
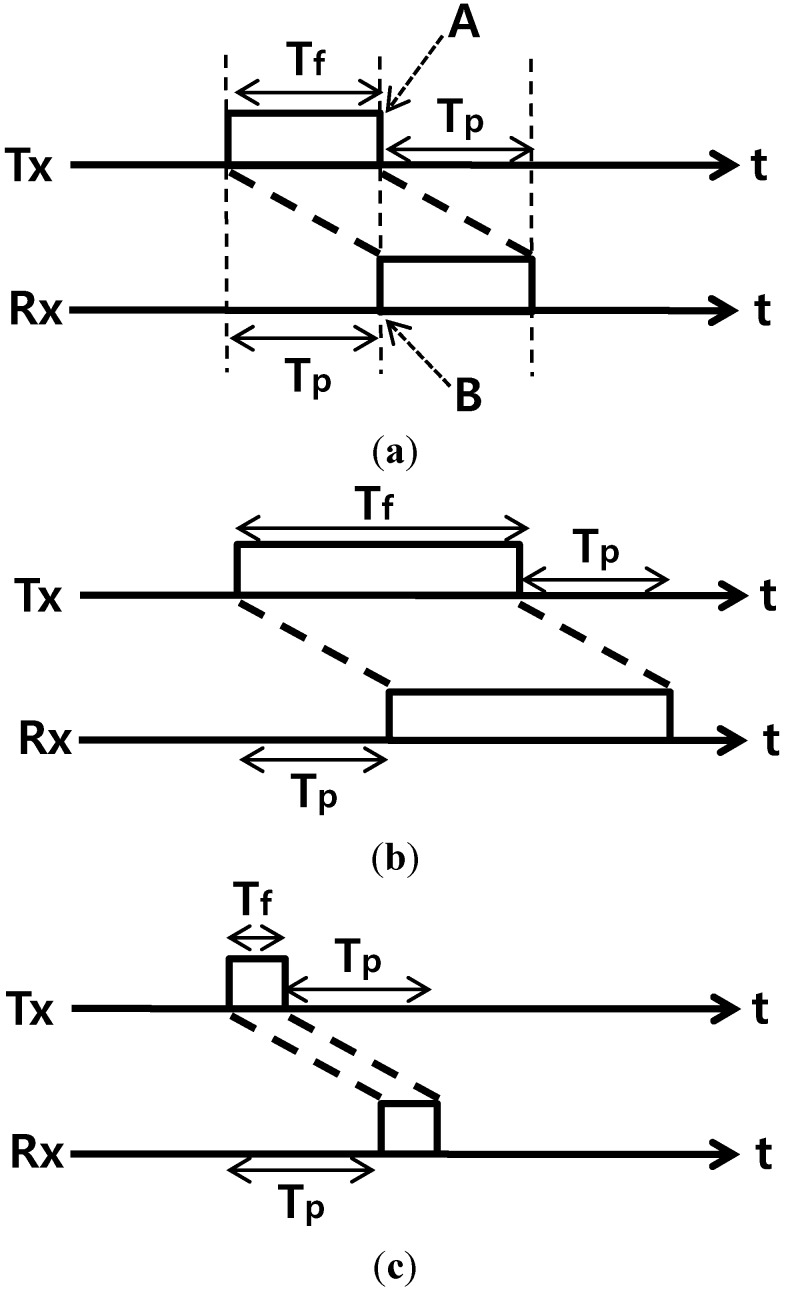
Frame timing between transmitting/receiving nodes. (**a**) *T_f_* *= T_p_*; (**b**) *T_f_* *> T_p_*; (**c**) *T_f_* *< T_p_*.

Each communication channel is assumed to be a shared common channel operating in a half-duplex mode. In underwater environments, the propagation delays are quite large, so the transmission/reception of neighboring nodes is restricted while a pair of omni-directional nodes is transmitting/receiving signals. That is, other nodes around the receiving node might not transmit signals to any node because interfering signals should not be given to the receiving node. In addition, other nodes around the transmitting node may not receive any other signal because the signals coming from the transmitting node become interfering signals. That is, if a pair of nodes transmit/receive signals, the time sections during which individual neighbor nodes may not transmit/receive signals will occur. The lengths of the node transmission times are regarded as frames to examine the three types of frame timing, as shown in Figure 2. *T_f_* is the frame time and *T_p_* is the one-way propagation delay. Figure 2a presents a case where *T_f_ = T_p_*, and because the last time point (A) of the frame in the transmission node is identical to the time point (B) of the beginning of the frame in the receiving node, the entire network can be made so that time slots in the length of *T_f_* are synchronized for all nodes. Frame-level synchronization among the nodes is assumed in the paper. As propagation delays between all nodes should be identical at *T_p_* to this end, equal spaces between the nodes should be assumed. That is, to apply the scheme proposed in this paper, one-dimensional underwater nodes are assumed to have been deployed linearly at equal spaces. Regarding the cases shown in Figure 2b (*T_f_* *> T_p_*) and Figure 2c (*T_f_* *< T_p_*), the complexity in management increases and the cases cannot be more excellent than the case shown in Figure 2a (*T_f_* *= T_p_*) in terms of maximizing the channel utilization by minimizing the idle sections on the time axis. That is, the scheme shown in Figure 2a is used in this paper. In underwater environments, a packet size is generally greater than *T_p_*. To match the inconsistency between a packet and a frame, a packet is segmented into the size, *T_p_*. On the other hand, concatenation is also applicable. Because the idle spaces between the time slots are removed through the operation in the form of time slots synchronized through Figure 2a, obtaining the maximum channel utilization simply becomes an issue of minimizing the idle time slot frequency.

**Figure 3:**
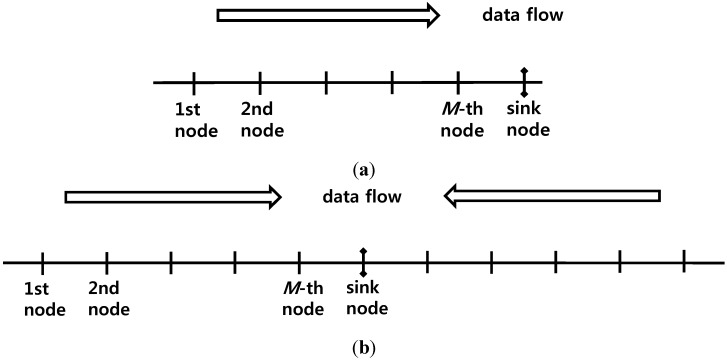
One-dimensional linear data flow. (**a**) The sink node is located at the end; (**b**) The sink node is located at the center.

Two one-dimensional linear node deployment and data flow models, as shown in Figure 3, are used. In these models, there is one sink node and if there are multiple sink nodes, the nodes can be divided based on the sink nodes into groups with a single sink node each. Figure 3b shows the cases where the sink node not located at the center are not considered because these cases are inferior to the cases where the sink node is located at the center. In cases where the sink node is located at the center, the total number of nodes shall be determined to be *N = 2M + 1* because cases where the total number of nodes is an odd number are more convenient. Transmitting the data gathered in the sink node is not considered here.

The case shown in Figure 3a is simpler than the case shown in Figure 3b because interference also comes from the opposite side of the sink node, in the case shown in Figure 3b. Therefore, a transmission/reception timing scheme that is good for the case shown in Figure 3b shall be found and applied to the case shown in Figure 3a. In the paper, the case shown in Figure 3b, which is more complicated, is analyzed. Let us find the methods for efficiently packing the time axis. Because each node is a half-duplex, when a node operates fully, it should spend a half of the operating time for transmission and the remaining half of the operating time for reception. That is, the maximum time efficiency for transmission is 1/2.

### 2.2. Proposed Schemes

**Figure 4:**
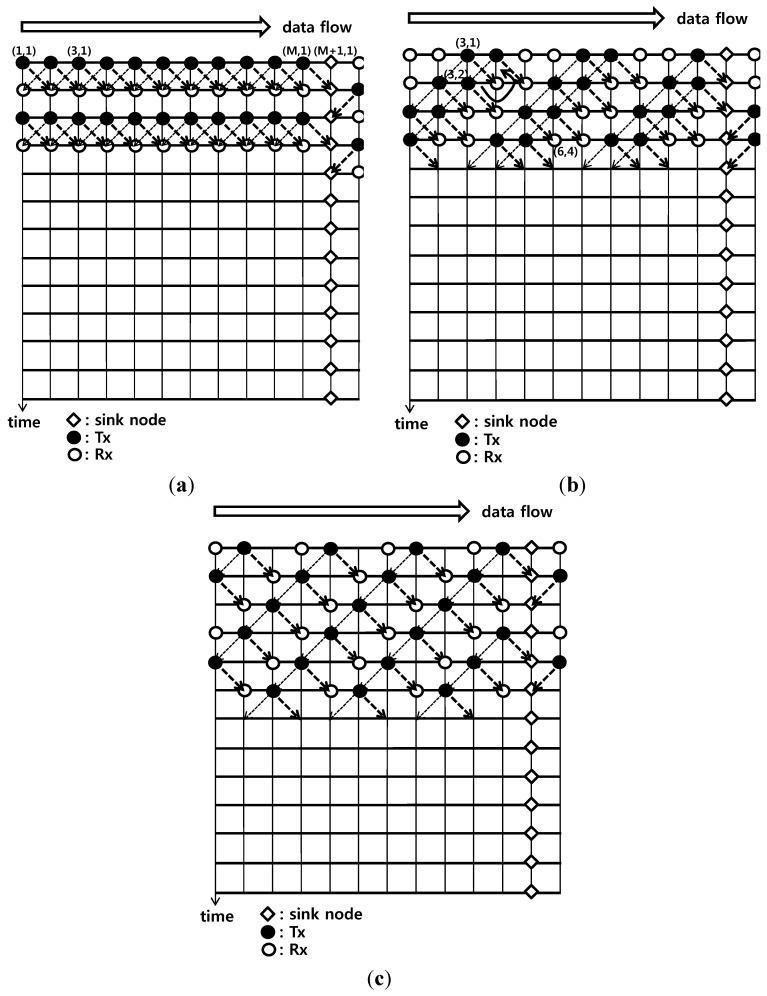
Proposed transmission/reception timing schemes. (The thick arrows represent data transfer) (**a**) Scheme 1; (**b**) Scheme 2; (**c**) Scheme 3.

In general, in Figure 4, the horizontal axis is the spatial dimension that indicates the nodes and the vertical axis is the time axis that indicates the time slots. Data flows from the left to the right until it arrives at the diamond shaped sink node and time flows from the top to the bottom. The filled circles indicate that the nodes are transmitting data and the empty circles indicate that the nodes are receiving data. When nodes are idle, there is no circle on the intersection points. For easy explanation, the individual intersection points are shown with the coordinates. (*i*, *j*) is the *j*th time slot from the top on the position of the *i*th node from the left. Owing to propagation delays, the transmitted frames are received by the neighboring nodes in the next time slot, which is indicated by the diagonal dotted lines in the figure. That is, the transmission from (*i*, *j*) is received by (*i* + 1, *j* + 1) and (*i* − 1, *j* + 1). The diagonal dotted lines originally progress infinitely, not just one box, and the power of the signals attenuates greatly as the signals progress. On the other hand, each dotted line is drawn in only one box for easy understanding of the figure.

Scheme 1 in Figure 4a shows the case where all nodes transmit data simultaneously and all nodes receive data simultaneously in the next time slot. From the standpoint of the receiving node (*i*, *j*), the data comes from (*i* − 1, *j* − 1), the immediate left upper box, and dominant interfering signals come from (*i* + 1, *j* − 1), the immediate right upper box. If the transmission power levels of the two transmissions are the same, the SIR should be approximately 1(=0 dB). Note that, according to the calculations in the following section, the transmission power levels are different by the node. Because the data is received from two directions, left and right in the sink node, the time points of receiving should be alternated.

Scheme 2 in Figure 4b is designed more elaborately. If the dominant interfering signals would not come from (*i* + 1, *j* − 1), the immediate right upper box from the standpoint of receiving node (*i*, *j*), the right upper box should not transmit data. Because the time axis use efficiency cannot be maximized if the box is idle, (*i* + 1, *j* − 1) should be receiving data. This can be explained in Figure 4b with examples of coordinates following the circular arrow. The transmission by (3, 2) becomes a reception by (4, 3) and because the transmission by (5, 2) will cause great interference, (5, 2) should be receiving data. If (5, 2) is receiving data, (4, 1) should be transmitting data. Because transmission by (*i*, *j*) requires transmission by (*i* + 1, *j* − 1), the transmission is located in the diagonal direction in the figure and the reception corresponding to the transmission is located one box away in the diagonal direction. Figure 4b shows the overlapping deployment of two of this pattern. Because all points are filled with filled or empty circles, the time efficiency has a maximum value of 1/2, as observed with Figure 4a. On the other hand, Figure 4b is more advantageous than Figure 4a in terms of the SIR because the dominant interference occurs on the left upper region at a distance of three boxes. For example, the frame to be received by (6,4) comes from (5,3) but the interference comes from (3,1). If an underwater path loss exponent of 1.5 is assumed for practical spreading [14], three times the difference in distance will be calculated as (1/3)^1.5^, indicating that the SIR gains of roughly 7.16 dB are provided. Because data is also received from two directions, left and right in the sink node in this case, the time points for receiving should be alternated to avoid a collision. In the paper, Scheme 2 in Figure 4b is proposed as a scheme that is the most time efficient and power saving.

Scheme 3 in Figure 4c is a scheme for a comparison with a lower time efficiency but more SIR gains. This is made by deploying the diagonal patterned designed, as shown in Figure 4b, and placing some empty areas. Because the idle areas account for 1/3 of all areas, the time efficiency decreases from 1/2 to 1/3 but the SIR gain becomes approximately 9.03 dB as the dominant interference occurs on the left upper region at a distance of four boxes.

## 3. Analysis and Numerical Results

### 3.1. SINR and Power Analysis

The volume of data originating in the individual nodes is assumed to be the same. Because the *i*th node cumulatively receives data from the first to (*i* − 1)th nodes, the amount received by the *i*th node is proportional to (*i* − 1). The amount of data transmitted by the *i*th node is proportional to *i* including the node’s data. Assume that the data rate for the transmission of each node is the transmission rate *C*. If *C_i_* is assumed to be the rate of data received from the *i*th node, *C_i_*=(*i* − 1)*C* (*i* = 2,3,4,…,*M* + 1). The SINR (signal-to-interference-plus-noise ratio) received by the *i*th node is assumed to be *SINR_i_*. The commonly used bandwidth is assumed to be *W* and the following Shannon capacity equation is approximately used:

(1) $$C_{i}\approx W\log_{2}\left(1+SINR_{i}\right)$$

where *C_i_* is the best achievable data rate. A higher *SINR_i_* or *C_i_* requires a higher modulation or a lower code rate in practical design due to the same transmission time for all nodes. Because the required *SINR_i_* that satisfies the data rate *C_i_* is calculated from Equation (1) and the actual *SINR_i_* should exceed or be equal to the calculated one, if the calculation is simplified with the bandwidth efficiency, *C_W_* = *C*/*W*, the following condition should be satisfied for the *SINR_i_*:

(2) $$\log_{2}\left(1+SINR_{i}\right)\geq \left(i-1\right)C_{W}$$

Note that because each node operates as a half-duplex, *C_W,avg_*, which is the average *C_W_*, becomes $\frac{1}{2}$*C_W_* in the case of Schemes 1 and 2 and $\frac{1}{3}$*C_W_* in the case of Scheme 3.

The SINRs at the individual nodes are calculated. Only the dominant interference and noise are considered approximately. Assume *P_i_* is the power received by the (*i* + 1)th node, which is transmitted from the *i*th node. Because the distance between two nodes and the attenuation in distance are always the same, the ratio of power *P_i_* to the transmitted power is always the same regardless of *i*. First, for Scheme 1 in Figure 4a, the SINR received by the *i*th node is obtained as follows:

(3) $$SINR_{i}=\frac{P_{i-1}}{P_{i+1}+P_{N}},\;i=2,3,\ldots ,M-1$$

where *P_N_* is the noise power. In most cases, the dominant interference comes from the box next to the right, as shown in Equation (3).

(4) $$SINR_{M}=\frac{P_{M-1}}{P_{M}{\left(\frac{1}{2}\right)}^{1.5}+P_{N}}$$

(5) $$SINR_{M+1}=\frac{P_{M}}{P_{M-1}{\left(\frac{1}{2}\right)}^{1.5}+P_{N}}$$

As a boundary condition, the dominant interference comes from the second box to the right for the node immediately before the sink node in Equation (4) and the sink node in Equation (5) (*P*_*M* + 2_ = *P_M_*, *P*_*M* + 3_ = *P*_*M* − 1_). The path loss exponent is assumed to be 1.5 [14]. Next, the *SINR_i_* for Scheme 2 in Figure 4b is as follows:

(6) $$SINR_{i}=\frac{P_{i-1}}{P_{N}},\;i=2,3$$

The first two SINRs received are shown by Equation (6) because they have only noises without any interference:

(7) $$SINR_{i}=\frac{P_{i-1}}{P_{i-3}{\left(\frac{1}{3}\right)}^{1.5}+P_{N}},\;i=4,5,\ldots ,M-1$$

In most cases, the dominant interference comes from the third box to the right as shown by Equation (7):

(8) $$SINR_{M}=\frac{P_{M-1}}{P_{M-3}{\left(\frac{1}{3}\right)}^{1.5}+P_{M-1}{\left(\frac{1}{3}\right)}^{1.5}+P_{N}}$$

(9) $$SINR_{M+1}=\frac{P_{M}}{P_{M-2}{\left(\frac{1}{3}\right)}^{1.5}+P_{M-1}{\left(\frac{1}{2}\right)}^{1.5}+P_{N}}$$

For the node immediately before the sink node in Equation (8), interference comes from the third box to the left and another interference comes from the third box to the right ($P_{M+3}=P_{M-1}$). As the dominant interference cannot be identified at this point, both are included in interferences. For the sink node in Equation (9), one interference comes from the third box to the left and another interference comes from the second box to the right ($P_{M+3}=P_{M-1}$). Next, the *SINR_i_* for Scheme 3 in Figure 4c is as follows:

(10) $$SINR_{i}=\frac{P_{i-1}}{P_{N}},\;i=2,3,4$$

The first three SINRs received are expressed as Equation (10) because they have only noises without any interference:

(11) $$SINR_{i}=\frac{P_{i-1}}{P_{i-4}{\left(\frac{1}{4}\right)}^{1.5}+P_{N}},\;i=5,6,\ldots ,M-1$$

In most cases, the dominant interference comes from the fourth box to the left, as shown by Equation (11):

(12) $$SINR_{M}=\frac{P_{M-1}}{P_{M-4}{\left(\frac{1}{4}\right)}^{1.5}+P_{M-1}{\left(\frac{1}{3}\right)}^{1.5}+P_{N}}$$

(13) $$SINR_{M+1}=\frac{P_{M}}{P_{M-3}{\left(\frac{1}{4}\right)}^{1.5}+P_{M-1}{\left(\frac{1}{2}\right)}^{1.5}+P_{N}}$$

Although Figure 4c is not precisely bilaterally symmetrical centering on the sink node, it is regarded as being approximately symmetrical and the SINRs on the left of the sink node are obtained. For the node immediately before the sink node in Equation (12), an interference comes from the fourth box to the left and another interference comes from the third box to the right ($P_{M+3}=P_{M-1}$). For the sink node in Equation (13), an interference comes from the fourth box to the left and another interference comes from the second box to the right ($P_{M+3}=P_{M-1}$).

The SINRs received by the individual nodes for Schemes 1–3 are obtained through Equations (3)–(13) above and these SINRs should satisfy Equation (2). Here, noise *P_N_* is considered to be 1 and the relative *P_i_*s are obtained based on the value. If the *SINR_i_*s are substituted into Equation (2), *P_i_*s will be obtained in sequence.

### 3.2. Numerical Results

**Figure 5:**
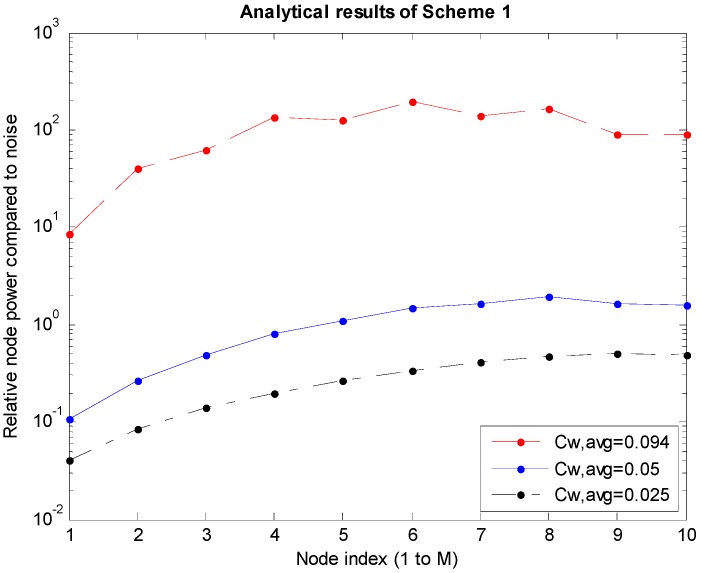
Power levels according to the node in Scheme 1.

**Figure 6:**
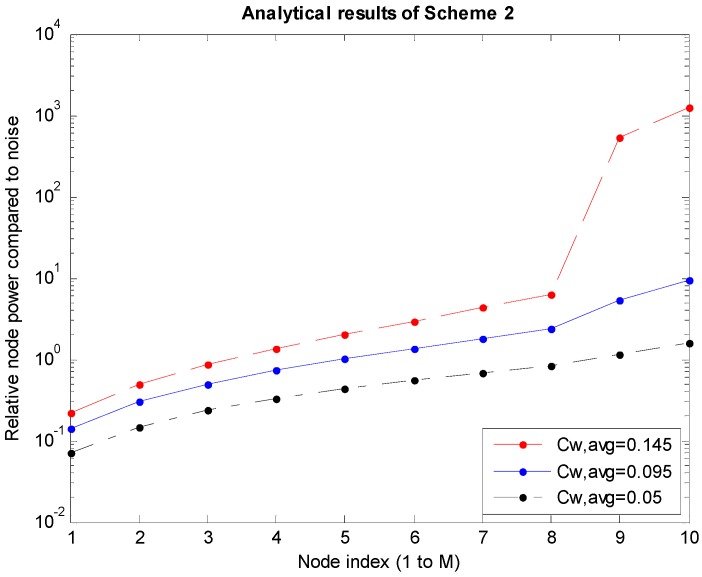
Power levels according to the node in Scheme 2.

**Figure 7:**
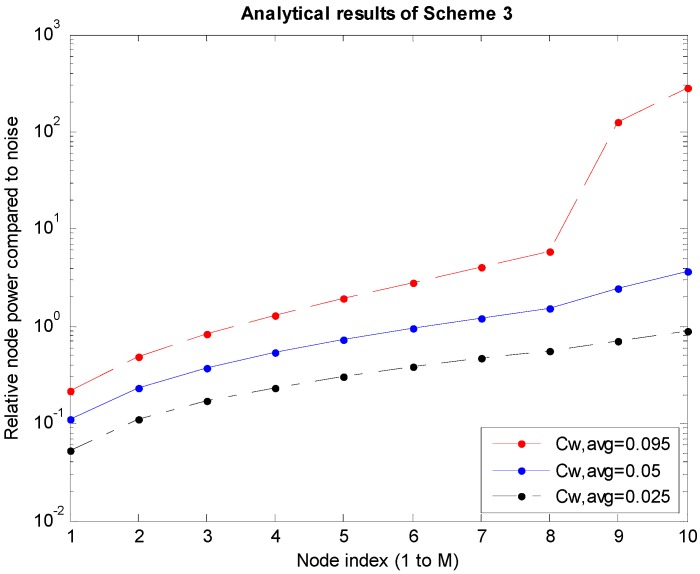
Power levels according to the node in Scheme 3.

*P_i_*s are the power levels of the nodes obtained through the analysis described in Subsection 3.1. The graphs shown in Figure 5, Figure 6 and Figure 7 are obtained for cases where *M* is 10, which is the number of nodes to the left of the sink node. In the graphs, *C_W,avg_* is the bandwidth efficiency, which is the transmission rate normalized to the bandwidth, and the system bandwidth efficiency is 2*MC_W,avg_* because there are *M* nodes each on the left and right of the sink node.

Figure 5, Figure 6 and Figure 7 correspond to Schemes 1–3, respectively, and the largest *C_W,avg_* value indicates the upper bound of transmission rates. In Scheme 1 (Figure 5), if *C_W,avg_* is higher than the upper bound, the SINR values (Equations (4) and (5)) of the boundary *M*th and (*M* + 1)th nodes will not satisfy the conditions under Equation (2). In Scheme 2 shown in Figure 6, when *P_M_*_-1_ increases infinitely in Equation (8), the upper bound of *SINR_M_* is 3^1.5^. Therefore, if *M* = 10 in Equation (2), the maximum value of *C_W_* will be determined to be 0.2924. Accordingly, the maximum value of *C_W,avg_* (=½*C_W_*) will become 0.1462. Similarly, in Scheme 3 shown in Figure 7, the same *C_W_* bound of 0.2924 is obtained using Equations (12) and (2). On the other hand, because the average *C_W,avg_* is calculated to be 1/3 of the *C_W_* bound, the actual bound is 0.0975. In Figure 6 and Figure 7, *P*_9_ and *P*_10_ become absurdly large near the upper bound of *C_W,avg_* because *P*_9_ (=*P_M_*_− 1_) should be much larger than the other terms in Equations (8) and (12) for a high SINR near the upper bound in Equation (2). Accordingly, *P*_10_ (=*P_M_*) should be also large in Equations (9) and (13) because of the large *P*_9_. Although the *C_W,avg_* value appears to be small, *C_W,avg_* is the transmission rate only at the first node and the transmission rate should be accumulated so that the transmission rate at the *M*th node, which is the last node should be *MC_W,avg_*. In addition, the power required by the nodes is generally larger in the nodes closer to the sink node.

**Figure 8:**
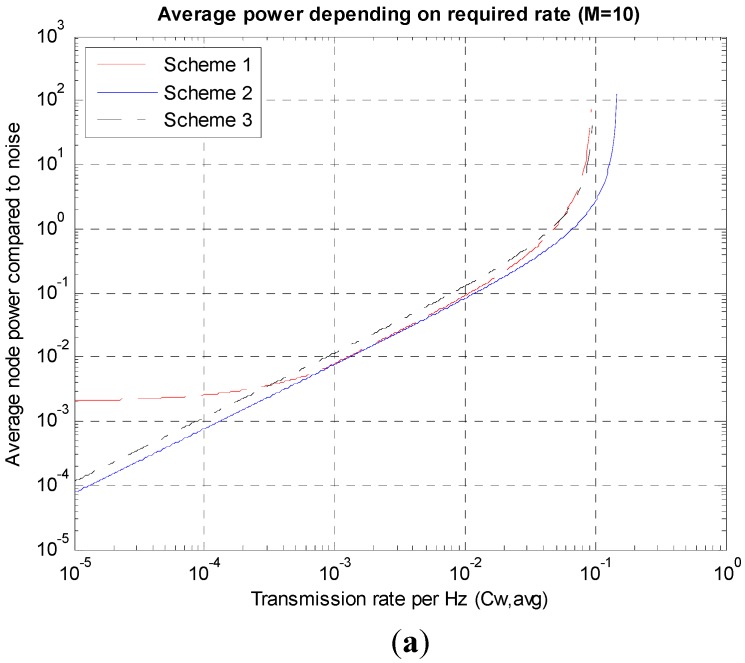

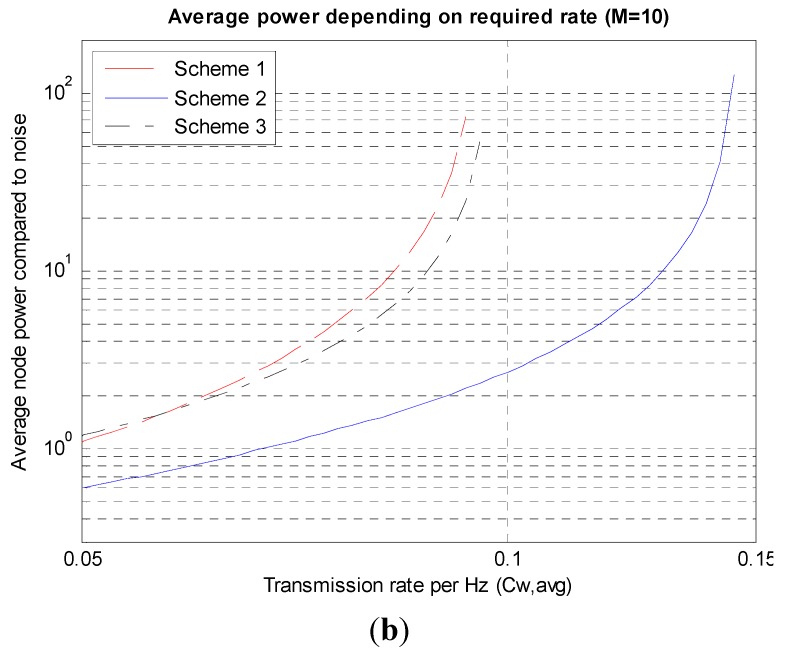
Average power depending on the transmission rates. (**a**) Wide range; (**b**) High rate range.

Figure 8 shows $\bar{P}$, the average of *P_i_*s obtained while changing the transmission rate *C_W,avg_* when *M* = 10 compared to the noise power ($\bar{P}=\frac{\sum P_{i}}{M}/P_{N}$). Figure 8a shows that the range of changes in the average power levels according to the changes in *C_W,avg_* values is very large, amounting to approximately 10^7^. To review the superiority among the Schemes, Scheme 2 is always the most excellent and the superiority of Schemes 1 and 3 changes according to *C_W,avg_*. Although the differences do not appear to be large in Figure 8a, if the differences are seen in the expanded figure, Figure 8b, Scheme 2 guarantees almost 50% higher transmission rates. The high *C_W,avg_* part in Figure 8b is expanded because this paper focuses on high rate environments.

**Figure 9:**
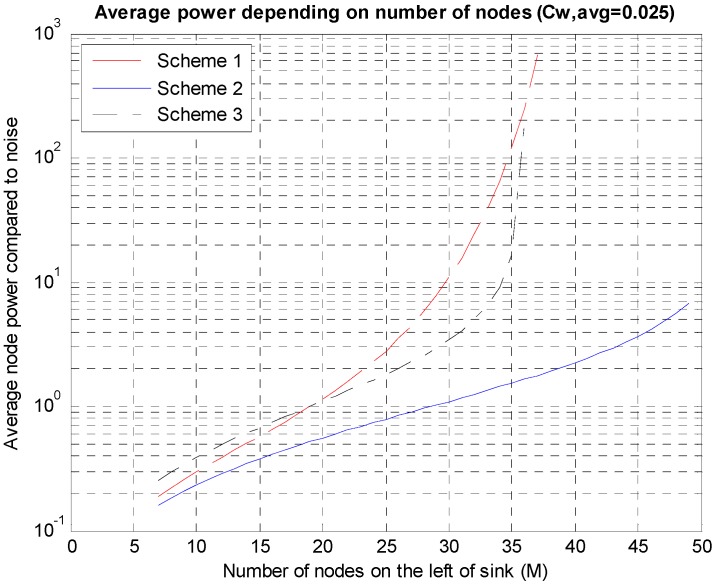
Average power levels according to the numbers of nodes.

Figure 9 shows the values of $\bar{P}$ obtained while increasing the number of nodes when the transmission rate *C_W,avg_* = 0.025. The average power level changes according to changes in the number of nodes by almost 10^4^ times. This is because as the number of nodes (*M*) increases, the amount of data to be transmitted by the nodes close to the sink node increases in proportion to *M*, leading to very high required SINRs. From the figure, Scheme 2 is the best and Schemes 1 and 3 cannot support high *M* values. For a fixed *C_W,avg_*, *M* is limited due to the limited *SINR_M_* in Equation (2). 

**Figure 10:**
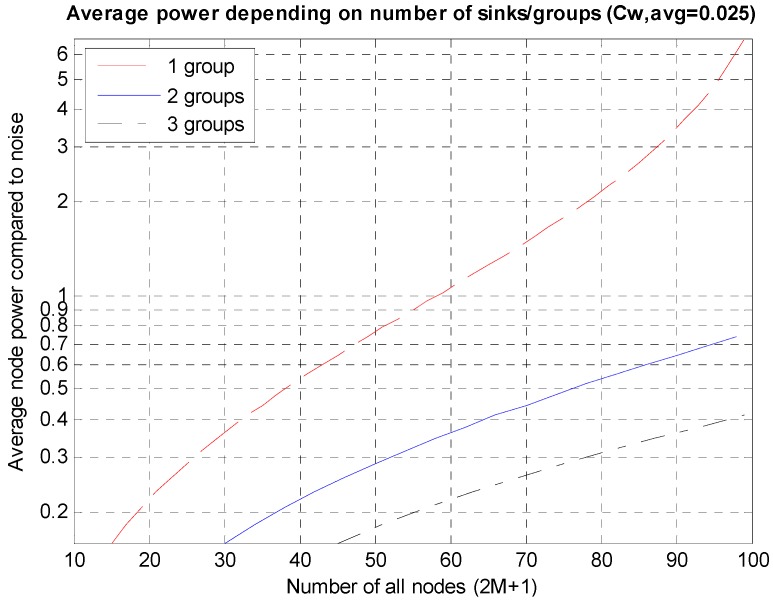
Decreases in the average power according to the network split.

As shown in Figure 9, as the number of nodes increases, the necessary average power rises rapidly. If the entire nodes are split into several groups and a sink node is deployed in each group, the necessary power can be reduced considerably. Because an environment where a sink node is deployed on the center, as shown in Figure 3b, is being analyzed, the entire nodes can be divided into multiple groups and a sink node can be deployed on the center of each group. The results are shown in Figure 10 in which, it can be seen that in the case of the three groups, the average power consumption decreases to 1/10 at the maximum compared to the case of a single group.

## 4. Simulation Results

### 4.1. Simulation Environments

Realistic underwater environments are reflected in the simulation. Parameters used in the simulation are listed in Table 1.

**Table 1:** Simulation parameters.

Parameter	Value
Number of sensor nodes (*M*)	10
Number of nodes including sink (*N*)	11
Internode distance (*D*)	100 m
Center frequency (*f_c_*)	6 kHz
Bandwidth (*f_BW_*)	2 kHz
Symbol rate (symbol/s)	2 ksps
Sound speed	1500 m/s
Frame size (*T_f_*)	0.067 s
Number of symbols in a frame	120 symbols
Transmit power limit	48 W (46.8 dBm)
Additive Gaussian noise	[14]
Path loss model	[15,16]
Data modulation	Uncoded QPSK
Target bit error rate (BER) for one hop	1%
Channel impulse responses	Generated based on East Sea of Korea

Each parameter is explained. Figure 3a is assumed to reduce the simulation load. The number of sensor nodes is 10 which is the same as the analysis section. The internode distance is 100 m, and the entire distance from the 1st node to the sink is 1 km. The center frequency and transmission bandwidth are 6 kHz and 2 kHz, respectively. The symbol rate is 2 ksps, which is the same as the bandwidth. The sound speed is assumed to be 1500 m/s, and the frame size is 0.067 s which is the time to travel 100 m. From the frame size and the symbol rate, the number of symbols in a frame is 134, but only 120 symbols are used for transmission and the remaining 14 symbols are reserved for the guard time to combat the delay spread. The maximum possible transmit power of a node is set to 48 Watts following the commercial limit of WHOI Micromodem-2 [17]. The maximum power ranges from 2.4 W to 100 W depending on the products [18]. The power of additive Gaussian noise is calculated through an integration over 5 kHz to 7 kHz in the power spectral density [14]. The path loss is based on Equations (1) and (2) of [15] with *k* = 1.5. The conversion from the electrical power to the acoustic one assumes the projector of [16].

The simulation does not contain channel encoding and decoding procedures because this simulation is not a link-level one but a system-level one. The uncoded QPSK is used for data modulation. The target uncoded BER is set to 1% considering the coded performance and acceptable simulation time. During a preliminary simulation with a raw QPSK symbol transmission without additive Gaussian noise, about 10% of the BER has occurred for the sink reception where the worst error rate is yielded because a real underwater channel in the simulation is very poor. This is similar to a binary symmetric channel (BSC) [19] with 10% error. However, an uncoded 10% error is too much for the coded performance. For example, a 10% error in BSC corresponds to an approximately 10% coded BER for (31, 11, 11) BCH code, which is commonly unacceptable. Less error in BSC results in much less coded BER. A 1% error in BSC yields down to 10^−7^ coded BER. An attempt is made to reduce the uncoded BER for the sink reception in a preliminary simulation, and the repetition and scrambling are applied for the transmission. A repetition of six times is adopted for the transmission from the 10th node to the sink. The original data is only 20 QPSK symbols among 120 symbols in a frame. As the data of this step is the union of data from the 1st node to the 10th node, the amount of data at the 1st node is only 1/10 of the last step from the 10th node to the sink. Therefore, repetition at the 1st node is 60 times to fill an entire frame. With a repetition of 6 times, about 0.03% error is yielded for the sink reception without Gaussian noise. Therefore, 1% BER is set considering the errors due to interference and noise. Scrambling is applied to avoid periodic interference and randomize it, and the combination of repetition and scrambling is similar to pseudo-noise code spreading in code division multiple access systems.

**Figure 11:**
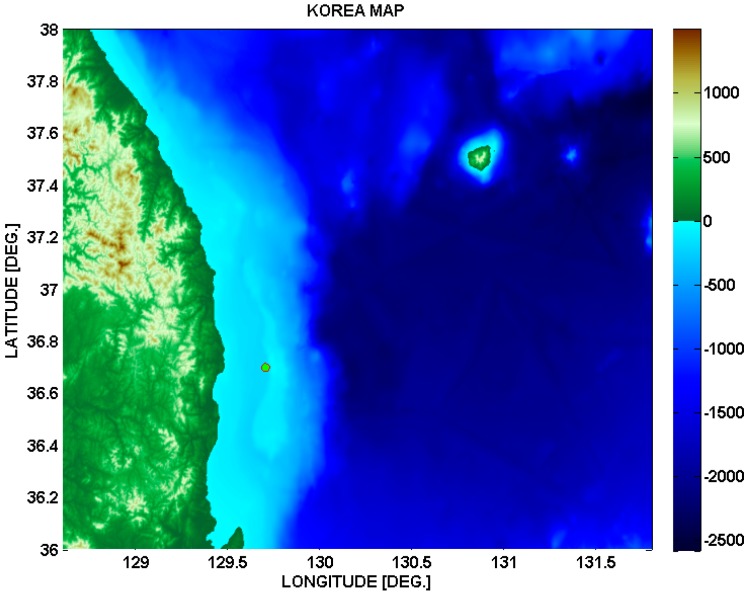
Target spot for underwater channels.

The channels are obtained by a Bellhop simulation using ray-tracing. The target spot is at a latitude of 36.7 and longitude of 129.7 in the East Sea of Korea, as shown in Figure 11. The average depth is 200 m on the spot. The transmitter and the receiver are located 2 m above the floor. Ten channel impulse responses are obtained for distances from 100 m to 1 km by 100 m. Table 2 gives an example of the relative power values of the delay taps for 100 m.

**Table 2:** Channel impulse response for a distance of 100 m.

**Time (s)**	0	0.0000676	0.225	0.227	0.229	0.231
**Relative Power (W/W)**	1	0.871	0.0522	3.17 × 10^−4^	3.20 × 10^−4^	1.95 × 10^−6^

**Figure 12:**
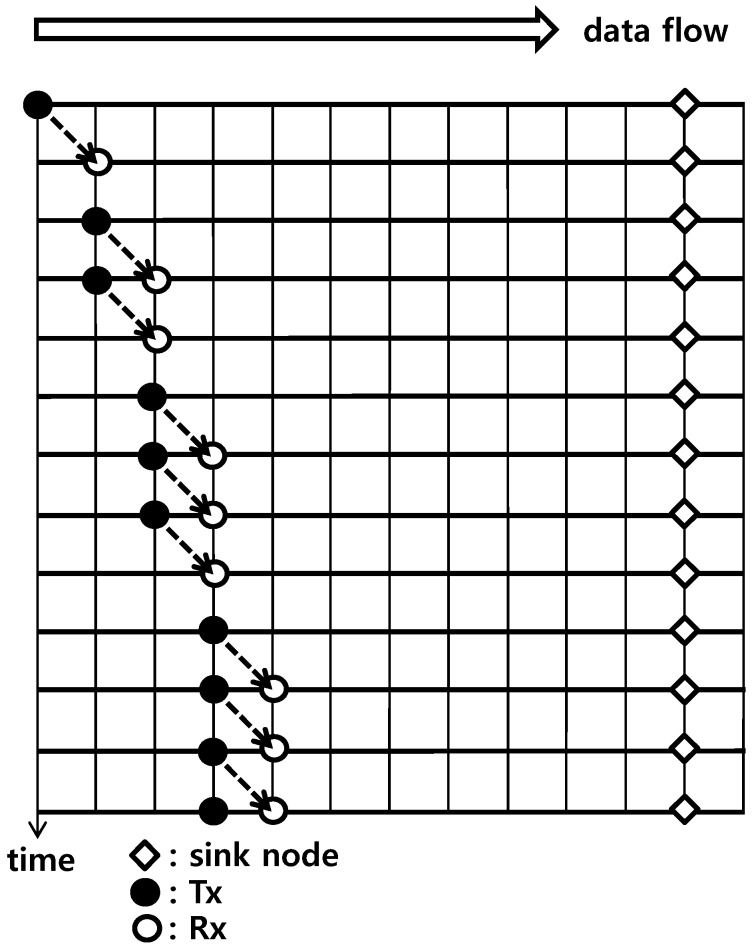
Transmission/reception timing of the packet train method.

In this section, the proposed Scheme 2 is compared with the packet train method because they have the same objective to enhance channel utilization. In the packet train method, each node relays data from the previous node to the next node, and the node transmits its own data simultaneously. In this scenario of data flooding to the sink, the amount of data to be transmitted is accumulated because relaying is processed toward the sink. Therefore, the number of frames for transmission proportionally increases with the node index in Figure 12. In the figure, the *i*th node uses *i* frames for transmission. Note that each node uses the same number of frames for transmission in Schemes 1–3, but the amount of data is different depending on the node. Therefore, if the last node (the 10th node) uses 100% of the frame capacity, the utilization of the 1st node can be said to be 10%. This is unavoidable because the amount of data is not the same. For the packet train method in Figure 12, there are many unused frames, neither for transmission nor for reception, but each transmission fully uses 100% of the frame capacity. For two schemes for comparison, the average utilizations are obtained. For the packet train method, 55 (=1 + 2 +…+ 10) data frames are transmitted for 65 (= 2 + 3 +…+ 11) time frames. The average is 55/65 = 0.846. For Scheme 2, 10 data frames are transmitted for two time frames, but the average utilization of a data frame is 55% (= (10% + 20% +…+ 100%)/10). Therefore, the average is 10/2 × 0.55 = 2.75. This means that the proposed Scheme 2 has 3.25 times more efficiency than the packet train method. For a fair comparison, a repetition of two times is applied to the packet train method because a nominal repetition of six times is used for Scheme 2. The difference in data rate is within 8.4%.

### 4.2. Simulation Results

**Figure 13:**
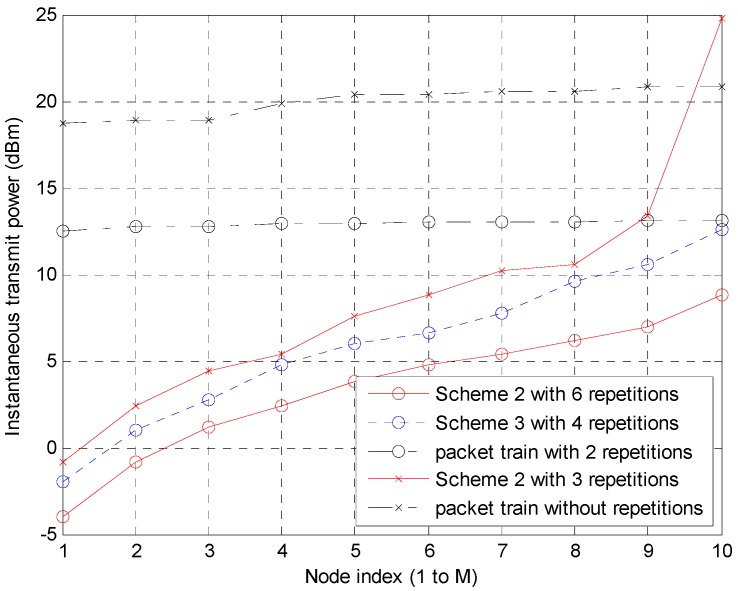
Transmit power of each node when the node is on the transmission state.

Figure 13 shows the transmit power at each node in Schemes 2 and 3 and the packet train method. The following eight scenarios in Table 3 are simulated. The former four provide a similar data rate to each other as so do the latter four.

**Table 3:** Simulation scenarios.

Scenario	Scheme
1	Scheme 1 with 6 repetitions
2	Scheme 2 with 6 repetitions
3	Scheme 3 with 4 repetitions
4	Packet train method with 2 repetitions
5	Scheme 1 with 3 repetitions
6	Scheme 2 with 3 repetitions
7	Scheme 3 with 2 repetitions
8	Packet train method without repetitions

Among the eight scenarios, scenarios 1, 5 and 7 do not satisfy BER below 1% criterion even without noise. Their worst BER values are 1.1%, 5.3% and 3.0%, respectively. Therefore, five curves are drawn in Figure 13. Scheme 2 with six repetitions is compared with Scheme 3 with four repetitions and the packet train method with two repetitions. Another comparison is between Scheme 2 with three repetitions and the packet train method without repetition. The power in Figure 13 presents an instantaneous value during a transmission frame. The abnormally high value at the 10th node of Scheme 2 with three repetitions is attributed to high interference from the surrounding active nodes. The 10th node suffers from a BER of 0.91% without noise. Therefore, the node is vulnerable to noise and the margin for noise is only 0.09%. Because the noise level is given, a high transmit power is required for the node. In addition, owing to the short inter-node distance of 100 m and achievable signal-to-noise ratio (SNR) for 1% BER, the transmit power level appears somewhat small between 0.4 mW to 300 mW. Overall, Scheme 2 outperforms the packet train method and Scheme 3 in terms of the instantaneous power, which could be a critical issue in cases where the maximum power is limited. In Scheme 2, the case of six repetitions performs better in terms of power consumption than that of three repetitions but the data rate becomes a half. One of the reasons for this is that the energy is doubly accumulated at the receiver because one information bit is doubly repeated at the transmitter. In other words, the case of six repetitions provides the same bit energy with only a half power. Therefore, 3 dB gain is achieved in Figure 13. The additional gain is from interference averaging. When repetitions of one information bit are summed at the receiver, the interference values are random because scrambling has been applied. Therefore, the summed interference values are not serious in most cases, and this phenomenon is similar to interference averaging in code division multiple access. For the above two reasons, the difference between Schemes 2 with six repetitions and with three repetitions is greater than 3 dB, as shown in Figure 13. The difference becomes greater for the packet train method.

**Figure 14:**
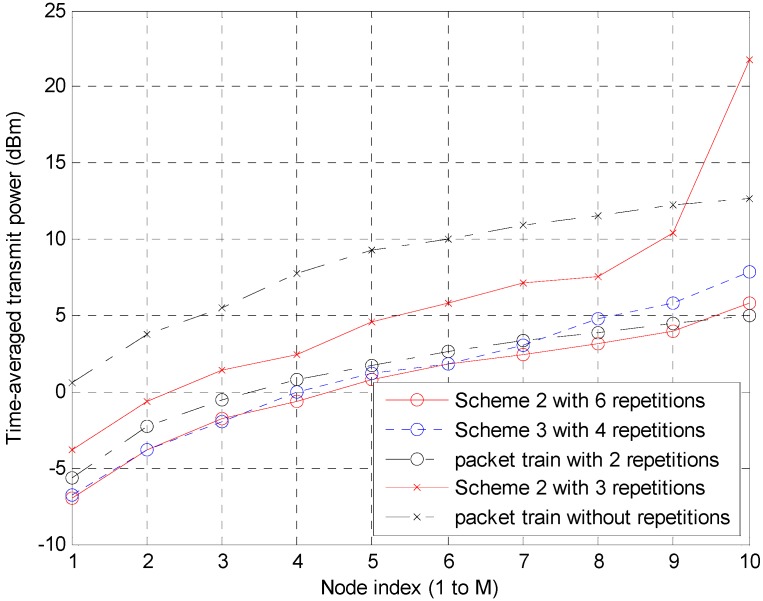
Time-averaged transmit power of each node.

On the other hand, in terms of battery life, the time average of the transmit power over the entire time period, including both the active and inactive states, is more important. Figure 14 shows the time average of each node’s transmit power. In case of Scheme 2 with six repetitions, compared to the packet train method with two repetitions, the power saving is clearer at the lower indexed nodes, even though performance degradation may occur at higher indexed nodes where data accumulation becomes heavier. In the case of Scheme 2 with six repetitions, compared to Scheme 3 with four repetitions, they show similar performance but Scheme 2 is slightly better. Because Scheme 3 cannot support two repetitions, Scheme 3, which is a variant of Scheme 2, is a good candidate in low interference environments (with many repetitions) but it cannot work in high interference situations (with a few repetitions.) Unlike the 6-repetitions case, Scheme 2 with three repetitions shows apparently better performance than the packet train method over the entire nodes except at the abnormal 10th node.

**Figure 15:**
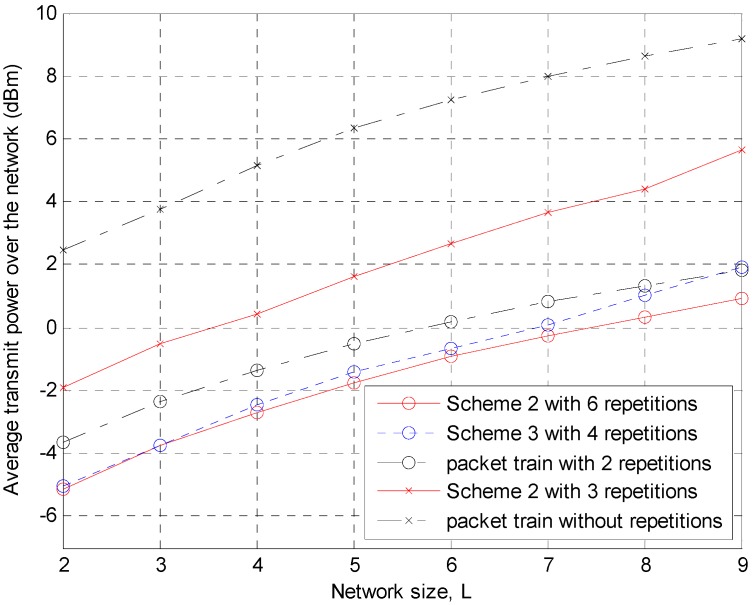
Average transmit power over the network depending on the number of nodes or the network size.

From Figure 14, it can be expected that if the abnormality caused by a heavy load cumulated at high-indexed nodes is removed, the superiority of Scheme 2 over the packet train method becomes clearer. Therefore, an attempt is made to reduce the network size, *L*, *i.e*., the number of nodes except the sink, to determine how much power saving gain can be achieved in smaller networks. Figure 15 shows the average value of the time-averaged power obtained in Figure 14 over all nodes from the 1st to the *L*th node. The power saving of Scheme 2 appears clear, particularly in the case of three repetitions.

**Figure 16:**
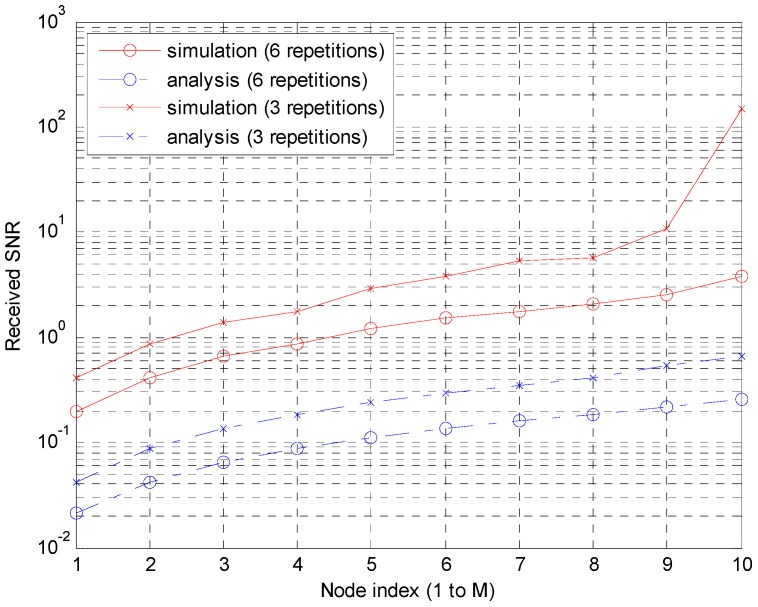
Power levels according to the node by the simulation and analysis in Scheme 2.

Figure 16 shows the signal to noise power ratio of Scheme 2 received at 100 m from the transmitter, comparing analysis and simulation results. Because the repetition is not modeled in the analysis, the equivalent data rate for the analysis is calculated. The equivalent *C_W__,avg_* values are 0.015 (= (120 symbols) × (2 bits/symbol)/(10 accumulations)/(6 repetitions)/*T_f_*/*f_BW_* × 50%) and 0.03 for six and three repetitions, respectively. The disagreement by almost 10-fold between the simulation and analysis is because the simulation is performed under much severer conditions, such as real channels with a delay spread, the inclusion of all interference, the absence of channel coding, and the use of guard time. On the other hand, the shapes of the curves agree well enough to support that the mathematical analysis in this paper based on a simple network topology is still useful in more realistic scenarios with different parameters.

## 5. Conclusions

The paper proposes three transmission/reception timing schemes that maximize the time axis use efficiency in one-dimensional linear sensor network environments to improve the resource efficiency for high rate transmission. Schemes 1 and 2 are those in which all nodes are transmitting or receiving data at all times with no idle time. Therefore, these schemes have the highest time use efficiency. Scheme 3 has the time use efficiency, which is 2/3 of the maximum time use efficiency but obtains more SIR gains. SINRs by node for the three schemes through mathematical analyses and power levels by node are derived based on the transmission rate conditions. In the numerical results, the power distributions by node, rate bounds, power levels depending on the rates and number of nodes, and network split gains are examined in a range of environments. Scheme 2 is found to be the best of the proposed schemes and can guarantee approximately 50% higher transmission rates at the same power in the numerical results. In the simulation results, Scheme 2 is compared with the existing packet train method. Scheme 2 outperforms the packet train method in transmission efficiency or power consumption but it has a limitation in network deployment geometry.
